# Genome-Wide Association Study Identifies 12 Loci Associated with Body Weight at Age 8 Weeks in Korean Native Chickens

**DOI:** 10.3390/genes12081170

**Published:** 2021-07-29

**Authors:** Jihye Cha, Hyojun Choo, Krishnamoorthy Srikanth, Seung-Hwan Lee, Ju-Whan Son, Mi-Rim Park, Nayeon Kim, Gul Won Jang, Jong-Eun Park

**Affiliations:** 1 Animal Genome & Bioinformatics, National Institute of Animal Science, RDA, Wanju 55365, Korea; wischa91@korea.kr (J.C.); tdpro@naver.com (J.-W.S.); cocci@korea.kr (M.-R.P.); ks901223@naver.com (N.K.); 2 Poultry Research Institute, National Institute of Animal Science, RDA, Pyeongchang 25342, Korea; hyojy@korea.kr; 3 Department of Animal Science, Cornell University, Ithaca, NY 14853, USA; srikanthkris@gmail.com; 4 Division of Animal and Dairy Science, Chungnam National University, Daejeon 34134, Korea; slee46@cnu.ac.kr; 5 Dairy Science Division, National Institute of Animal Science, RDA, Cheonan 31000, Korea; kwchang@korea.kr

**Keywords:** Korean native chicken, genome-wide association study, body weight, gene set enrichment analysis

## Abstract

Meat from Korean native chickens (KNCs) has high consumer demand; however, slow growth performance and high variation in body weight (BW) of KNCs remain an issue. Genome-wide association study (GWAS) is a powerful method to identify quantitative trait-associated genomic loci. A GWAS, based on a large-scale KNC population, is needed to identify underlying genetic mechanisms related to its growth traits. To identify BW-associated genomic regions, we performed a GWAS using the chicken 60K single nucleotide polymorphism (SNP) panel for 1328 KNCs. BW was measured at 8 weeks of age, from 2018 to 2020. Twelve SNPs were associated with BW at the suggestive significance level (*p* < 2.95 × 10^−5^) and located near or within 11 candidate genes, including *WDR37*, *KCNIP4*, *SLIT2*, *PPARGC1A*, *MYOCD* and *ADGRA3*. Gene set enrichment analysis based on the GWAS results at *p* < 0.05 (1680 SNPs) showed that 32 Gene Ontology terms and two Kyoto Encyclopedia of Genes and Genomes pathways, including regulation of transcription, motor activity, the mitogen-activated protein kinase signaling pathway, and tight junction, were significantly enriched (*p* < 0.05) for BW-associated genes. These pathways are involved in cell growth and development, related to BW gain. The identified SNPs are potential biomarkers in KNC breeding.

## 1. Introduction

Growth traits are one of the most economically important characteristics in the chicken industry and are greatly associated with both the breeding value and retail meat value [1]. Therefore, identification of genetic determinants that affect growth traits in commercial chickens is fundamental to their industrial breeding. Growth traits are quantitative characteristics that are affected by various genes; therefore, it is hard to make rapid and efficient improvements using conventional selection methods [2].

Korean native chickens (KNCs) are known to have originated in the Korean Peninsula more than 1400 years ago. Under the Korean government-led KNC restoration project, five distinct lines have been established based on their feather color. These lines, namely, White (Baeksaek Jaerae-jong), Black (Heuksaek Jaerae-jong), Grey (Hoegalsaek Jaerae-jong), Yellow (Hwanggalsaek Jaerae-jong), and Red (Jeokgalsaek Jaerae-jong), have been registered with the Domestic Animal Diversity Information System of the Food and Agriculture Organization (http://dad.fao.org/, accessed on 7 November 2020) [3]. Owing to its characteristic flavor and texture, meat from KNCs is preferred by domestic consumers over that from commercial broilers [4]; however, the slow growth performance and high variation in the BW of KNCs remain an issue [5].

With recent improvements in high-throughput next-generation sequencing technologies, it has become possible to uncover genomic regions that are highly associated with specific traits at the genome-wide level. In particular, a genome-wide association study (GWAS) is the most effective method to determine genomic loci significantly associated with quantitative traits. A previous GWAS has uncovered important genomic regions affecting the growth, reproduction, and disease-resistance traits in chickens [6]. Furthermore, GWASs have been increasingly used to map quantitative trait loci (QTL) for economic traits, such as body weight (BW), in chickens. Although many previous GWASs have reported BW-associated single nucleotide polymorphisms (SNPs) and QTL across the genome, in most cases, chromosomes 1 and 4 were found to be strongly associated with BW at different ages in various breeds of chickens [7,8,9,10].

Several studies have already been carried out on QTL that affect economic traits, such as meat quality and BW, in KNCs [11,12]; however, a further GWAS, based on a large-scale KNC population, is needed to identify underlying genetic mechanisms related to growth traits. Therefore, in our study, a GWAS was performed to identify potential genomic SNP markers and candidate genes associated with the BW trait in a population of 1328 KNCs to establish important biological pathways affecting BW in KNCs.

## 2. Materials and Methods

### 2.1. Animals and Phenotypes

A total of 1328 birds from two KNC breeds, namely, Red (*n* = 732) and Yellow (*n* = 596), were collected from 2018 to 2020. All the chickens were provided by the Poultry Research Institute of the National Institute of Animal Science (NIAS). Chickens (*n* = 10) were randomly assigned to windowless pens (size: 0.5 × 1.0 m) under standard conditions of temperature, humidity, and ventilation, with automatic ventilation and temperature control during the experimental period. Feeds were provided separately from 0 to 6 weeks of age (CP 19%, ME 2850 kcal) and 7 to 8 weeks of age (CP 14.5%, ME 2820 kcal) according to the nutritional requirements of each growth stage. Chickens had free access to water during all growth stages. All phenotypic data related to BW were measured at age 8 weeks. In this study, BW at age 8 weeks was chosen as the only major target trait owing to the Korean Native Chicken (KNC) breeding strategy. Our poultry breeding program has been focused mainly on improvement of market-weight in KNCs. Currently, KNCs have been shipped to the market at age 10 weeks; therefore, it is important to develop an efficient breeding strategy that can ensure that individuals with good growth capability can be selected in advance before reaching market-weight. The related research to determine the age at which to select birds for breeding programs showed that BW at age 8 weeks had the highest correlation among early growth periods with market-weight [13]. Moreover, BW at age 8 weeks was significantly positively correlated not only to market weight but also to BW at age 20 weeks; thus, it is considered the best representation of the overall growth of KNCs. All procedures followed relevant guidelines formulated by the Institutional Animal Care and Use Committee of the NIAS (approval number NIAS 20181278).

### 2.2. Genotyping and Quality Control

Genomic DNA was isolated from the 1328 blood samples using the Wizard genomic DNA purification kit (Promega, Madison, WI, USA). After measuring the DNA concentration and purity using a NanoDrop 1000 spectrophotometer (Thermo Fisher Scientific, Wilmington, DE, USA), the total DNA samples were genotyped using the 60 K chicken SNP chip (Illumina, San Diego, CA, USA) involving 57,636 SNPs. SNPs located on the sex chromosomes (Z and W) and those with unknown positions were excluded from this analysis. The Plink software was used to perform SNP filtering for the genotypic data based on the following criteria [14]: minor allele frequency <1%; low genotyping call rate, <90%; missing genotype calls >10%; and Hardy–Weinberg equilibrium at *p* < 0.00001. After quality control, 1328 animals and 33,878 SNPs were deemed eligible for subsequent analysis.

### 2.3. Principal Component Analysis (PCA)

Principle component analysis (PCA) was performed to estimate the genetic relatedness between individuals and to account for potential population stratification prior to the GWAS. PCA based on the genomic relationship matrix (GRM) was performed to calculate the eigenvectors for all genotypic data using the genome-wide complex trait analysis (GCTA) tool [15].

### 2.4. Genome-Wide Association Analysis, Heritability, and Variance Component Estimation

Genome-wide association study (GWAS) was carried out between all the genotyped SNPs and BW using a mixed linear model (MLM). The MLM for BW was performed using the breed (Red or Yellow), sex (female or male), and year of hatching (2018, 2019, or 2020) as fixed effects and the top two PCs as covariates. All association tests were performed using the MLMA option in GCTA, based on the following model (Equation (1)):

*y* = *Xb* + *Zµ* + *g* + *e* (1)

where, ***y*** is the vector of phenotypic values for the BW; *X* and *Z* are incidence matrices for the vectors for parameters ***b*** and ***µ***, respectively; ***b*** is a vector of fixed effects including the breed, sex, and year of hatching; ***µ*** is the vector of the additive genetic effect of the candidate SNP to be tested for association; ***g*** is the accumulated effect of all the SNPs captured by the GRM; ***e*** is the vector of the residual effect. The significance threshold for the GWAS was defined using the Bonferroni correction method. We set the following two thresholds for our data: *p* < 1/N for suggestive significance and *p* < 0.05/N for a 5% genome-wide significance level, where N is the number of the SNPs remaining after quality control. The genomic inflation factor (λ) was calculated using the CMplot package in R with the median option. Manhattan and quantile–quantile (QQ) plots were created using the CMplot package in the R software [16].

Estimation of variance components was calculated using the restricted maximum-likelihood analysis option in GCTA, whereas genomic heritability (*h*^2^) was calculated as the ratio of the additive genetic variance (Vg) to the phenotypic variance (Vg *+* Ve).

### 2.5. SNP Annotation and Gene Set Enrichment Analysis

Gene set enrichment analysis (GSEA) was performed based on the GWAS result to identify the biological pathways related to a group of genes harboring significant BW-associated SNPs. First, SNPs to be used for functional analysis were detected by filtering *p*-values < 0.05 based on the GWAS results. Using the SnpEff version 4.3 software [17], the filtered SNPs were annotated to genes if they were within the gene region or within a flanking region 5 kb upstream or downstream to the gene. The latest version of the *Gallus gallus* (chicken) genome assembly 6 was used as the reference genome. When we first performed the GSEA based on the GWAS data, we referred to several published studies that have reported relevant similar analyses [18,19,20,21,22,23,24]. Based on these related reports, the *p*-value threshold for filtering GWAS SNPs to be used in GSEA differed between 0.05 and 0.005 among reports. Furthermore, the criteria for setting the flanking region when performing gene annotation, which also differed among reports, were either 5 or 15 kb. Thus, we adjusted the criteria for selecting GWAS SNPs to be used in GSEA to include approximately 1000 unique genes in the final dataset, resulting in the final SNP filtering criteria being determined as *p* < 0.05 and a 5 kb flanking region. Annotated genes were submitted to the Database for Annotation, Visualization and Integrated Discovery (DAVID) for Gene Ontology (GO) and Kyoto Encyclopedia of Genes and Genomes (KEGG) analyses [25,26]. We decided to use an EASE cutoff of 0.1 as the default option when submitting the gene list to the DAVID tool for GSEA. The EASE score refers to the modified fisher exact *p*-value and is calculated by penalizing (removing) one gene within the given category from the list and calculating the resulting Fisher exact probability for that category [27]. Based on the EASE score, the enrichment *p*-value in the Functional Annotation chart was calculated, and we defined the *p* value threshold (*p* < 0.05) for significantly enriched GO/KEGG term [20,21].

## 3. Results

### 3.1. Descriptive Statistics of Phenotype and Heritability

**Figure 1:**
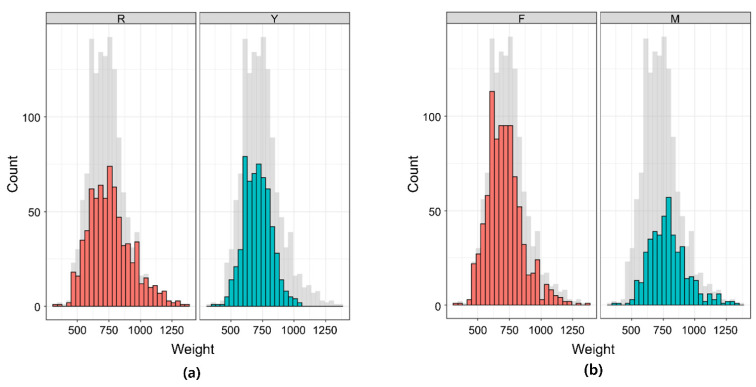
Phenotypic distribution of the body weight records measured at age 8 weeks. (**a**) Distribution for each breed (R: Red Korean native chicken (KNC); Y: Yellow KNC); (**b**) Distribution for each sex (F: female; M: male) The shadow in the background represents the total population.

A descriptive statistical summary for BW in the 8-week-old KNC population is provided in Table 1. The median BW values showed that the Red breed was heavier than the Yellow breed and that males were heavier than females. It is obvious from Figure 1 that the total bodyweight records, as well as those sorted by breed and sex, had an approximately normal distribution. The genomic heritability for BW showed a high estimate of 0.47, as shown in Table 2.

### 3.2. PCA

**Figure 2:**
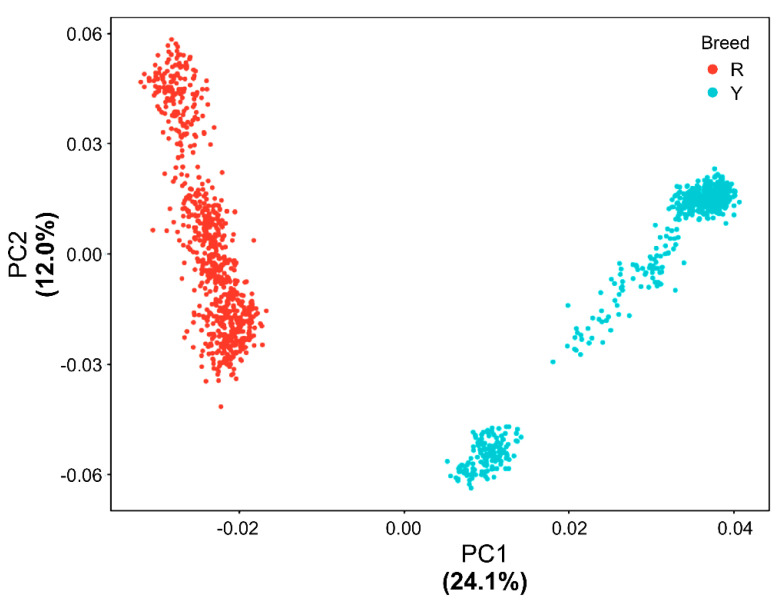
Population structure of two Korean native chicken (KNC) breeds based on the principal component (PC) analysis. Dots of different colors represent each individual from the two breeds (R: Red KNC; Y: Yellow KNC).

Figure 2 shows the first two PCs of the GRM. The first PC explained 24.1% of the total variance and clearly separated the Red and Yellow breeds. However, the two breeds were not separated by the second component, which explained 12.0% of the total variance. Therefore, we treated the first two PCs as covariates and included them within the GWAS model as fixed effects to adjust for variations in the population structure.

### 3.3. GWAS

**Figure 3:**
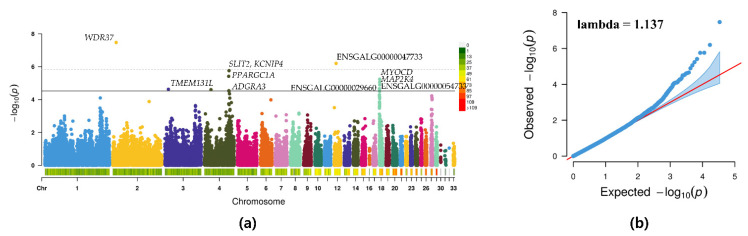
(**a**) Manhattan plots of mapped single nucleotide polymorphism (SNP) markers associated with body weight. Each dot corresponds to a SNP within the data set. The dot color represents the chromosome on which the SNP is located; the dot position represents the −log_10_-transformed *p*-value of the SNP. The horizontal dotted line denotes the genome-wide significance threshold level (−log_10_(*p*) > 5.83); the solid line denotes the suggestive threshold level (−log_10_(*p*) > 4.53). (**b**) Quantile–quantile plot of the genome-wide association study.

The GWAS was performed for 33,878 SNPs to find genomic regions associated with the BW of KNCs at 8 weeks of age. The MLM-based GWAS revealed that 12 SNPs were associated with the BW and reached the suggestive significance level (*p* < 2.95 × 10^−5^) (Table 3). Manhattan and QQ plots of the BW are shown in Figure 3. The significant BW-associated 1, 1, 5, 1, and 4 SNPs were located on each chromosome 2, 3, 4, 12, and 18, respectively. Of the top SNPs detected, the most significant SNP Gga_rs15062501, located on chromosome 2, is a novel SNP that has not been previously reported in the chicken QTL database. This SNP was identified to be an intron variant of the gene *WDR37*, encoding a member of the WD-repeat protein family. On chromosome 4, which harbored the largest number of significant BW-associated SNPs, the SNP GGaluGA265847 is located within the gene *KCNIP4*; Gga_rs14490865 is in an intron region of the gene *SLIT2*; GGaluGA265650 is located within the gene *PPARGC1A*; Gga_rs15508929 is located in the upstream region of the gene *TMEM131L*; and GGaluGA265746 is located in the downstream region of the gene *ADGRA3*. Other suggestive regions were detected on chromosome 18, where two SNPs (Gga_rs14105952 and Gga_rs13506093) are located in the genes *MYOCD* and *MAP2K4*, respectively.

### 3.4. Gene Set Enrichment Analysis

To further investigate significant GO/KEGG pathway terms associated with BW, gene set enrichment analysis was performed for the group of genes to which the SNPs were mapped. A total of 1680 SNPs showed an association with BW at a nominal threshold of *p* < 0.05, and these SNPs were mapped to 931 unique genes (Supplementary Table S1). Subsequently, gene set and pathway analyses were performed using DAVID. A total of 51 GO terms, including 39 biological processes and 12 molecular functions, and five KEGG pathways were enriched in the mapped genes (Supplementary Table S2). The most significantly enriched (*p* < 0.05) GO terms and KEGG pathways are shown in Table 4. The most significant GO terms were positive regulation of transcription from RNA polymerase II promoter (GO:0045944), motor activity (GO:0003774), transcriptional activator activity, RNA polymerase II core promoter proximal region sequence-specific binding (GO:0001077), and receptor signaling protein serine/threonine kinase activity (GO:0004702). The KEGG pathway analysis showed that the genes harboring significant BW-associated SNPs were highly enriched (*p* < 0.05) in two pathways, which included the mitogen-activated protein kinase (MAPK) signaling pathway and tight junction.

## 4. Discussion

In this study, we performed a GWAS to detect significant SNPs associated with BW in a population of 1328 KNCs and explored genomic regions around these SNPs for candidate genes.

Several studies [28,29,30] have reported higher BW records in KNCs at age 8 weeks than those obtained in this study, which could be attributed to differences in the number of animals surveyed, in nutrition during the growth period, or in the methods of measurement; however, the fundamental reason could be genetic differences among the breeds. While commercial broilers are intensively bred for high BW and rapid growth, indigenous chickens, including KNCs, generally have delayed growth [31], which is thought to be the cause of the large gap in BW records at the early age of 8 weeks.

The moderate heritability estimate of 0.47 was quite similar to those in various studies that have reported heritability estimates of BW at different growth stages in a range from 0.15 to 0.55 in different native chicken breeds [20]. Moreover, the estimate was also in agreement with the results of Cahyadi et al. [8], who estimated genetic parameters for growth-related traits in KNCs and showed that the BW at age 8 weeks had a genetic heritability of 0.45.

The GWAS result showed that 12 SNPs were associated with BW at the suggestive significance level (*p* < 2.95 × 10^−5^) and were located near or within 11 candidate genes, including *WDR37*, *KCNIP4*, *SLIT2*, *PPARGC1A*, *MYOCD*, and *ADGRA3*. BW is closely related to the growth of various tissues, including muscle, fat, and bone. The candidate genes identified in this study are correlated to the growth and development of these tissues. Of the top SNPs detected, the most significant, Gga_rs15062501, is located on chromosome 2 and is an intron variant of the *WDR37* gene, encoding a member of the WD-repeat protein family, which is involved in the growth-related processes, including cell cycle progression and gene regulation. Although this variant is a noble SNP that has not yet been reported in the chicken QTL database, Khatri et al. [32] reported that *WDR37* was differentially expressed between broilers selected for rapid and slow growth.

Some previous GWASs conducted in different populations of chickens have reported that chromosome 4 is the critical region that influences growth traits, particularly BW [33,34,35]. Consistently, our results showed that chromosome 4 harbored the largest number of significant BW-associated SNPs. One of the top SNPs located on chromosome 4 was detected in *KCNIP4.* This potential candidate gene belongs to a family of potassium channel-interacting proteins, which have extensive physiological functions, including neurotransmitter release, smooth muscle contraction, heart rate adjustment, and insulin secretion. *KCNIP4* has also been identified as an important QTL region associated with growth traits in a different chicken population [28]. The candidate gene *SLIT2* on chromosome 4 interacts with proteins that affect cell adhesion and movement in embryonic development, influence the activity of growth factors, and even modulate their own activities [36]. Pértille et al. [37] reported that *SLIT2* is strongly associated with back yield, feet weight, and fat traits in chickens. Additionally, Liu et al. [38] reported *SLIT2* as a potential gene associated with muscle weight in an F2 chicken population. The identified candidate gene *PPARGC1A* on chromosome 4 is involved in the chemical processes related to lipid and energy production in addition to adipocyte differentiation and regulation of mitochondrial biogenesis [39]. *PPARGC1A* is expressed in abdominal fat tissue in broilers [40] and has been reported to be a potential selection marker against abdominal fat in chickens [41]. The candidate gene *ADGRA3* on chromosome 4 belongs to the gene family encoding adhesion G protein-coupled receptors, which facilitate cell adhesion and are involved in numerous developmental processes, such as neural, cardiovascular, immune, and endocrine development [42,43]. Lyu et al. [44] discovered that *ADGRA3* harbored a functional mutation affecting chicken growth in an inbred chicken population.

Other suggestive regions were detected on chromosome 18 and included, in particular, the *MYOCD* gene, which is transiently expressed in skeletal muscle progenitor cells of somites and functions as a molecular switch for muscle differentiation [45]. Furthermore, *MAP2K4* encodes a member of the MAPK family, which is involved in various cellular processes, such as cell proliferation, differentiation, transcription regulation, and development [46]. This gene has been found to be differentially expressed between chicken populations selected for low or high residual feed intake [47,48].

Functional analysis based on less significant SNPs derived from GWAS results not only detects SNPs that are not significantly associated but are still likely to affect phenotype variation but also helps understand the complex mechanisms among genes and related pathways, which can be involved in growth performance [23]. The gene set enrichment analysis based on the GWAS results revealed that many of the BW-associated genes were enriched in GO terms, with the largest number of genes involved in the GO term “positive regulation of transcription from RNA polymerase II promoter” (GO:0045944). This term is related to any process that activates or increases the frequency, rate, or extent of transcription from the RNA polymerase II promoter. Development and growth of eukaryotic organisms require proper regulation of gene expression, and RNA polymerase II is the key component in this process [49]. Therefore, an increase in transcription from the RNA polymerase II promoter leads to an increase in cell growth and development, which is closely related to BW gain. Motor activity (GO:0003774), which was another significantly enriched GO term, involves unique myosin isoforms, including MYH1A, MYH1B, MYH1D, MYH1E, and MYH1F. These myosin isoforms play a vital role related to muscle fiber regeneration and repair for muscle development [50].

The KEGG pathway analysis showed that genes harboring significant BW-associated SNPs were remarkably enriched in the MAPK signaling pathway (gga04010). The MAPK signaling pathway is an important regulator of skeletal muscle development and was found to be activated during differentiation of a myogenic cell line [51]. MAPK signaling has been reported to serve an essential role in a variety of differentiation processes, including adipogenesis, neurogenesis, and myogenesis [52]. Moreover, MAPK provides a link between myogenic transcription factors that directly activate muscle genes and chromosomal remodeling activities related to muscle differentiation [53]. In addition to the MAPK signaling pathway, tight junction (gga04530) was identified as a highly enriched KEGG pathway associated with the BW trait. Tight junctions are a type of cell–cell junction that form and maintain cell adhesion [54]. The process of interaction between adjoining cells and the extracellular matrix are key components in the regulation of gene expression and cell development [55].

## 5. Conclusions

To the best of our knowledge, this is the first study to explore genetic markers and biological pathways associated with BW using 60K SNP chip data in a population of more than 1000 KNCs. This GWAS identified a total of 12 BW-associated SNPs, which were located near or within 11 candidate genes, including *WDR37*, *KCNIP4*, *SLIT2*, *PPARGC1A*, *MYOCD*, and *ADGRA3*. The main biological role of the identified candidate genes was found to be the regulation of cell growth and development, which can affect BW. Our findings provide insights into BW-associated genes and pathways. Furthermore, the identified SNPs can potentially be used as biomarkers in KNC breeding.

## Figures and Tables

**Table 1:** Descriptive statistics for body weight in two Korean native chicken (KNC) populations at age 8 weeks.

Breed	Number of			Record of (g)			
	Total	Males	Females	Max	Min	Mean	SD
	1328	441	887	1380	335	738.22	150.11
Red KNC	732	234	498	1380	335	761.99	173.66
Yellow KNC	596	207	389	1050	370	709.05	108.05

SD: Standard deviation.

**Table 2:** Results of variance component estimation for body weight.

Source	Variance	SE
Vg	5842.90	768.40
Ve	6625.94	401.97
Vp	12,468.84	645.38
Vg/Vp	0.47	0.04

SE: Standard error; Vg: Additive genetic variance; Ve: Environmental variance; Vp: Phenotypic variance.

**Table 3:** Top single nucleotide polymorphisms (SNPs) associated with body weight trait identified using a linear mixed model approach.

SNP ID	Chr	Position	Minor Allele	Major Allele	MAF	*p*-Value	SNP Effect	Gene	Location
Gga_rs15062501	2	10469206	G	A	0.28	3.37 × 10^−8^	33.67	*WDR37*	Intron
GGaluGA083256	12	6388108	C	A	0.23	6.33 × 10^−7^	−28.50	ENSGALG00000047733	Intron
Gga_rs14490865	4	75155441	G	A	0.21	1.73 × 10^−6^	33.42	*SLIT2*	Intron
GGaluGA265847	4	74925016	G	A	0.14	1.75 × 10^−6^	40.33	*KCNIP4*	Intron
GGaluGA265650	4	74010712	A	G	0.09	3.85 × 10^−6^	46.22	*PPARGC1A*	Intragenic
Gga_rs14105952	18	893043	G	A	0.34	5.80 × 10^−6^	27.70	*MYOCD*	Intragenic
Gga_rs13506093	18	804322	A	G	0.47	8.70 × 10^−6^	−25.80	*MAP2K4*	Downstream
Gga_rs13506254	18	1185022	C	A	0.28	1.27 × 10^−5^	26.63	ENSGALG00000054733	Intron
Gga_rs15809279	18	353546	G	A	0.46	2.17 × 10^−5^	−22.30	ENSGALG00000029660	Intron
Gga_rs13503427	3	10517429	G	A	0.32	2.35 × 10^−5^	24.12	−	Intergenic
Gga_rs15508929	4	19924807	G	A	0.05	2.48 × 10^−5^	−40.57	*TMEM131L*	Upstream
GGaluGA265746	4	74486766	A	G	0.14	2.89 × 10^−5^	33.16	*ADGRA3*	Downstream

Chr: Chromosome; MAF: Minor allele frequency; SNP effect: the β coefficient indicating the SNP effect size estimated for the minor allele.

**Table 4:** Gene Ontology (GO) terms and Kyoto Encyclopedia of Genes and Genomes (KEGG) pathways significantly enriched (*p* < 0.05) for genes associated with body weight.

Category	Term_ID	Term	Count	%	*p*-Value	Genes
KEGG_PATHWAY	gga04010	MAPK signaling pathway	21	3.0043	0.00	*MAP2K3*, *MAP2K4*, *MAP3K3*, *TGFB2*, *IL1R1*, *BDNF*, *NFATC3*, *MAPK8IP3*, *TGFBR1*, *MAPK8IP1*, *PPP3CA*, *FGF7*, *TAOK1*, *GNA12*, *MKNK2*, *RAC2*, *MAPT*, *SOS1*, *SOS2*, *MAP4K3*, *MAP4K4*
KEGG_PATHWAY	gga04530	Tight junction	9	1.2876	0.03	*PPP2R2B*, *MYH1E*, *MYH1F*, *MYH1D*, *PPP2R2A*, *MYH1A*, *MYH1B*, *AMOTL1*, *MYH10*
GOTERM_MF_DIRECT	GO:0003774	Motor activity	6	0.8584	0.01	*MYH1E*, *MYH1F*, *MYH1A*, *MYH1B*, *MYH10*, *MYO1F*
GOTERM_MF_DIRECT	GO:0001077	Transcriptional activator activity, RNA polymerase II core promoter proximal region sequence-specific binding	13	1.8598	0.02	*ARNT2*, *MYOCD*, *PLAG1*, *NFATC3*, *EBF2*, *NRF1*, *HIF1A*, *MEOX1*, *ELF1*, *NFIA*, *TBX20*, *TP63*, *ZNF750*
GOTERM_MF_DIRECT	GO:0004702	Receptor signaling protein serine/threonine kinase activity	7	1.0014	0.02	*MAP3K3*, *MAP2K4*, *TGFB2*, *BMPR2*, *TAOK1*, *TGFBR1*, *MAP4K4*
GOTERM_BP_DIRECT	GO:0045944	Positive regulation of transcription from RNA polymerase II promoter	34	4.8641	0.01	*RB1*, *RNASEL*, *BMPR2*, *PID1*, *KDM1A*, *TNKS*, *PLAG1*, *GATA4*, *TCF20*, *LDB2*, *MYSM1*, *HIF1A*, *NPAS2*, *ABRA*, *SPIC*, *PPP3CA*, *EPCAM*, *CREB3L1*, *TBX20*, *CYTL1*, *PPARGC1A*, *E2F7*, *TP63*, *NCOA1*, *XRCC6*, *AUTS2*, *LMO4*, *DAB2IP*, *ARNT*, *EBF2*, *ASH1L*, *BMP5*, *NFIA*, *CDH13*
GOTERM_BP_DIRECT	GO:2001235	Positive regulation of apoptotic signaling pathway	5	0.7153	0.01	*DAB2IP*, *PTEN*, *CASP2*, *TP63*, *TGFBR1*

%: the percentage of genes associated with particular annotation terms in the total input gene list; MF: Molecular function; BP: Biological process.

## Data Availability

The data presented in this study are available on request from the corresponding author.

## Supplementary Materials

The following are available online at https://www.mdpi.com/article/10.3390/genes12081170/s1, Table S1: List of all body weight-associated single nucleotide polymorphisms (SNPs) (*p* < 0.05) used for gene set enrichment analysis, Table S2: List of all Gene Ontology terms and Kyoto Encyclopedia Genes and Genomes pathways enriched for body weight-associated genes.
